# Observations on the Proposed Tax on Entering the Profession

**Published:** 1830-10-01

**Authors:** 


### TAX ON ENTERING THE PROFESSION.

A sharp fire has lately been kept up between medici and mediculi respecting an apprehended additional tax on those who are entering the profession of medicine and surgery. The law is about to establish a tax, we understand, of about two hundred pounds on all those who are about to start in the pursuit of forensic wealth and fame. The reason alleged is the crowded state of the legal ranks, and the consequent deterioration, not only of the trade, but of the respectability of the members themselves. A redundancy of population has, in all countries where it exists, led to deterioration of morals and the commission of crimes. It cannot be otherwise. Poverty is the parent of vice — and necessity is the mother of invention—not the invention of good for our neighbours, but of the means of filling our own bellies, no matter at whose expense. China furnishes a striking example of super-abundance of population. A bad harvest puts millions to death by hunger and leads to terrible scenes of cruelty. But at all times it authorises the crime of infanticide—or what is equivalent to infanticide, exposure of infants on the rivers and in the streets. This crime against nature is *permitted* on account of the surplus of population. So, when a profession or trade becomes over-stocked, it leads to all kinds of vile tricks as well as to ingenious inventions. If it only produced generous and fair competition, all would be well; but he can have only a very small share of discernment, who does not see the host of disingenuous arts which flow — unavoidably flow, from redundancy of hands. MEDICULUS,* in censuring any measure that can check the influx of members into the medical profession, seems to overlook entirely this view of the subject—and to anticipate nothing but the improvement of medical science by the collision of rivalry, and the competition for bread. Alas! he knows little of the means and of the machinery which are called into operation in medical competition! The labour of improving medical science would stand a very poor chance, when opposed to the art of extending medical connexions, not by inventing more successful modes of treatment than our neighbours, but by devising more ingenious methods of traducing our competitors and aggrandizing ourselves at their cost! When MEDICULUS grows up in time, to the size of MEDICUS, he will see a little of this, both in town and country.

What is the remedy? Nothing, we conceive, can check the increase of the evil, but a diminution of the inordinate influx of members into the great professional family—whether legal or medical. We certainly cannot admire a direct money-tax on aspirants, propos-

* Medical Gazette, July 3, 1830.

ed, of course, as a prohibition against the introduction of the indigent into our ranks. Such a measure operates against *merit* as well as against indigence, and deserves all the execration which Mediculus has bestowed on it. But let the tax be laid on in the shape of KNOWLEDGE—and we think it could scarcely be too heavy. This is the proper way to reduce the redundancy of the medical population. It would act in a doubly beneficial way—by lessening the number of candidates and by increasing the scale of their education, which increase would render them more liberal as well as more respectable. Thus, without prescribing this or that seat of learning, the proofs of classical and other elementary learning should be as ample as those required for the highest grades of the profession emanating from our universities. If the examinations in Greek, Latin, mathematics, and all the arts and sciences that adorn life, were as rigid for one member or class as for another, then we would have real and proper equality in our ranks, as well as a much smaller number. The discontent and jealousy engendered by different grades and invidious distinctions would be done away with at once. And we prophecy that the necessity of placing this strong barrier of high acquisition on the multitude of labourers who rush towards the harvest of medical practice, will soon be felt and seen by those who have the legislative regulations in their department. Indeed the process is, at this moment, going forward for an amelioration of the profession, in the gradual elevation of scale by which medical and surgical competency is measured. The corporate bodies should unite, and agree on one large and liberal regulation respecting medical education. The check to redundancy, on this plan, does not prohibit the poor man because he has not money, but because he has not education and knowledge—nor does it admit the rich man's son because he has wealth, but because he has a proper degree of information. To sum up in one sentence:—Equalization of titles and rank, in the republic of medicine, can only be based on equalization of scholastic education aud scientific acquirements. This primary equilibrium established — this common starting-post fixed, talent, assiduity, friends—CHANCE, will still afford ample sources of inequality in public estimation and fortune, which we can no more regulate by legislative enactments, than we can control the clouds that float along the Heavens, or the "wind that bloweth where it listeth."

---

